# Updates on digital mental health interventions for children and young people: systematic overview of reviews

**DOI:** 10.1007/s00787-025-02722-9

**Published:** 2025-04-25

**Authors:** Shaun Liverpool, Ciarán Mc Donagh, Julie Feather, Chinebuli Uzondu, Michelle Howarth, Fariba Bannerman, Axel Kaehne, Celeste Foster, Ceu Mateus

**Affiliations:** 1 Edge Hill University, Ormskirk, UK; 2 Transformative Transport Services Design Initiative (TRATSEDI), London, UK; 3 University of Salford, Salford, UK; 4 Lancaster University, Lancaster, UK

**Keywords:** Child, Adolescent, Mental health, Technology, Implementation, Sustainability

## Abstract

Digital mental health interventions (DMHIs) are increasingly recommended for children and young people (CYP) as a promising way to prevent and treat mental health problems. Here, we summarised and consolidated findings from existing systematic reviews to provide an overview of what is known, and which areas need further investigation. Systematic searches were conducted until January 2024 using PubMed, PsycINFO, MEDLINE, CINAHL, Scopus and Google Scholar. Records were screened against predefined criteria and quality assessed using A MeaSurement Tool to Assess Systematic Reviews (AMSTAR-2) and Joanna Briggs Institute (JBI) Critical Appraisal Checklist for Systematic Reviews. A study protocol was co-developed with key stakeholders and registered on the Open Science Framework. From 941 records, 51 systematic reviews published between 2000 and 2023 of generally moderate quality, targeting CYP 0 to 25 years, were included in our narrative summary. DMHIs were delivered in a variety of ways, including online video calls, apps and various combinations, underpinned mostly by cognitive behaviour therapy. DMHIs supported different mental health problems, but mostly symptoms of anxiety and/or depression. Although generally effective, some studies reported mixed results with limited evidence when focusing on longer-term outcomes. Other benefits of DMHIs included reduced costs and time investments for families, and increased accessibility and acceptability of support. Practitioner preparedness and unclear ethics/safety measures were identified as factors impacting engagement and potential effectiveness. The findings suggest that DMHIs can be a valuable tool for supporting CYP. However, realising the full potential of DMHIs for all CYP may require more high-quality research utilising DMHIs that are diverse in theoretical underpinnings and target audiences.

**Supplementary Information:**

The online version contains supplementary material available at 10.1007/s00787-025-02722-9.

## Introduction

Currently, there is a mental health crisis facing children and young people (CYP). In the last decade, mental health concerns amongst CYP have reached a critical stage [1–5], with a global prevalence of disorders reported at 10% [6]. Recent statistics from the United Kingdom (UK) revealed that one in five CYP between the ages of eight and twenty-five years are likely to experience symptoms of a probable mental health disorder [7]. Several factors including wars and civil unrest [8, 9], climate change [10] and the global pandemic [11] have contributed to this decline in CYP’s mental health. As a result, mental health services are stretched beyond their capacity and are not capable of attending to the needs of CYP [12]. The significant waiting times further compound the problem, leaving CYP to experience worsening symptoms, such as suicide ideation or attempts, that require immediate and urgent care [13, 14].

There are various forms of mental health support available to CYP including school-based interventions, community-based services and healthcare-based psychotherapeutic programmes [15–17]. However, alongside waiting times, other barriers such as financial constraints, social stigma and geographical accessibility are notable limitations to face-to-face support [18]. In response to these limitations and the COVID-19 pandemic, policymakers and practitioners have recognised the need for more accessible mental health services, resulting in a noticeable uptake in digital-based and technology-enhanced services, commonly referred to as digital mental health interventions (DMHIs) [19, 20].

Increasingly, DMHIs have shown significant promise in bridging the demand-to-access gap to support CYP [21, 22]. Numerous public healthcare organisations, government bodies and charities have also championed this approach to CYP’s mental health care and support [23]. These recommendations are generally based on the increasing numbers of CYP having access to at least one smart device which can enhance the delivery of DMHIs through mobile applications and virtual reality experiences [24]. Correspondingly, there is a large body of research showing that DMHIs are effective in addressing and preventing mental health disorders in CYP [25–27].

Despite the wealth of existing evidence and plethora of available DMHIs, reviews have predominantly explored the effectiveness of DMHIs on specific mental health conditions like anxiety and depression or focused on specific types of DMHIs like online cognitive behavioural therapy programs [26, 28–32]. Lehtimaki et al. [32] also highlighted the limited number of evidence-based DMHIs for CYP 10 to 24 years. Broader explorations have discussed the diverse, multifaceted impact of digital technology on CYP, including its impact on academic performance, social interactions and overall mental and physical health [33–35]. Although these insights contribute to the ongoing usage and rapid development of new DMHIs, less is known about the factors that contribute to the successful uptake, implementation, sustainability and subsequent effectiveness of DMHIs in a real-world context [32].

Successful implementation of DMHIs has been inconsistent due to challenges faced when transitioning from research to practice [36, 37]. More specifically, the complex interaction between patients, professionals, organisations, and policies has introduced several barriers [38]. These include the absence of resources, lack of training, and ethical concerns. To address these barriers, implementation frameworks that capture acceptability and usability are recommended to provide a more systemic approach to the development and implementation of DMHIs [36, 39–41]. However, rapid technological innovations and a need for cultural adaptations to DMHIs call for continuous research to inform the scale-up of DMHIs and the refinement of existing frameworks. More information is also needed to enhance standards for the sustainability of DMHIs [41]. Without this, several DMHIs will be discontinued when initial funding ends [37, 42]. Therefore, attention to sustainability during the implementation phase of DMHIs is a growing recommendation, as policymakers and funders aim to allocate resources effectively and efficiently [43].

A key priority of this review was to ensure that sufficiently robust evidence informs the current and future needs of CYP who can benefit from DMHIs, as well as the decision-making of those who commission or deliver such services. This umbrella review also sets out to build on the important findings by Lehtimaki et al. [32] by reviewing the evidence before, during and after the Covid-19 pandemic and for a wider age range. Therefore, the overarching aim is to synthesise evidence from existing systematic reviews to explore what is known, and which areas need further investigation, to enhance uptake, implementation and sustainability of effective DMHIs for CYP from early years to early adulthood. In so doing, the following research questions (RQs) were addressed:

1. What is the range, scope and quality of evidence from systematic reviews on DMHIs for CYP with (or at risk of) mental health problems?
2. Who are DMHIs being offered to and for what mental health symptoms/problems?
3. What digital formats are commonly used for DMHIs, and what are the common underpinning therapeutic theories?
4. Are DMHIs effective in improving the mental health and well-being of CYP, and are there any other benefits?
5. What are the key factors to consider that may support or limit engagement and potential effectiveness?

## Methods

### Study design and protocol registration

An overview of systematic reviews, commonly referred to as an umbrella review [44], was conducted with guidance from the Cochrane Overview of Reviews handbook and the Joanna Briggs Institute’s recommendations [45, 46]. An umbrella review was selected due to its ability to provide a clear and comprehensive overview of the existing evidence on our topic [45]. The findings were reported according to the preferred reporting items for overviews of reviews (PRIOR) checklist [47] (Online Resource 1) and the review protocol was co-produced in October 2023, and registered on the Open Science Framework in June 2024, during the data extraction and quality appraisal phase [48]. Changes to protocol included rephrasing of the research questions and analytical approach undertaken.

### Databases and search strategy

The search strategy (Online Resource 2) was developed and piloted in collaboration with a Specialist Librarian (FB). The searches were conducted using EBSCOhost platform for PsycINFO, MEDLINE, CINAHL, Scopus, PubMed (National Library of Medicine) and Google Scholar on 6th October 2023 and updated on 2nd January 2024. Handsearching, expert consultations and reference pearling were used to manually identify additional relevant records.

### Eligibility criteria and selection process

After deduplication on the Rayyan systematic review software [49], three reviewers (SL, CU, CMD) were involved in the screening and article selection process moving from titles and abstracts to full texts. The eligibility criteria were first piloted during team meetings and then independently applied by at least two reviewers. Any disagreements about a study’s eligibility were resolved through discussions with a third member of the review team. Studies were included in this overview of reviews on the following basis:

(I) If they broadly explored the effectiveness of DMHIs that aimed to improve the psychological well-being of CYP. This review categorised CYP as young people up to, and including, age 25 years as this age group is at increased risk of mental health problems [6]. Inclusion criteria required at least 50% of the studies in the identified reviews to be within this age threshold. As guided by our community engagement and involvement activities (described below), eligible reviews needed to include at least one study targeting children below the age of 18 years. This decision was based on the opinions that reviews targeting only young adults—18 to 25-year-olds (e.g., university students)—may not be representative of younger children’s developmental stages, experiences and outcomes. Reviews where age was undefined but the age range for CYP was implied, such as school-aged or youth, were also included.
(II) If they focused on any digital tools used in mental health promotion, prevention and treatment. These included but were not limited to websites, mobile applications, online games or consoles and computer-assisted programmes, robots and digital devices, virtual reality, and mobile text messaging or social media networks. Interventions that were offered hybrid and included an in-person element were also considered.
(III) If they focused on mental health, mental well-being and/or mental health conditions. These included common mental health problems (diagnosed or undiagnosed) like anxiety and depression as well as factors/symptoms related to mental health conditions such as loneliness, low mood and suicidality. Reviews focused on co-morbid conditions including physical health conditions were beyond the scope of this review.
(IV) If they followed any type of systematic-style review of primary empirical studies. Therefore, reviews including scoping reviews, with a transparent, reproducible method, rigorous search strategy and predetermined criteria were eligible. Narrative reviews without a systematic search approach or other reviews of reviews were excluded.
(V) If published between January 2000 and January 2024. This approach was adopted to capture the most relevant, up-to-date research reflecting contemporary theories, methodologies, and advancements in the field while maintaining relevance to current practice and policy.
(VI) If accessible in full-text and English format. This approach was adopted to ensure consistency in data interpretation and reduce the risk of translation bias.

### Quality appraisal

To assess the rigour and quality of the included reviews, reviews that included randomised or non-randomised studies, or both were appraised using the AMSTAR-2 checklist, A MeaSurement Tool to Assess systematic Reviews [50]. The AMSTAR-2 is a valid and reliable 16-item quality appraisal tool that assesses the methodological quality of systematic reviews. AMSTAR-2 is also a widely recognised tool that ensures quality assessments are consistent with international standards. Responses to each item were categorised as “yes” to include those that partially addressed the criteria and “no” for those reviews that did not meet or apply to the criteria. The four critical domains assessed in the identified reviews were, (1) registering a protocol, (2) performing an adequate search strategy, (3) justifying the excluded studies, and (4) conducting a publication bias assessment. Similar to other studies we adopted a scoring system [51]. If the answer was “yes” to all four domains the study was categorised as high quality, if two or three were “yes” then moderate quality and if only one or none were answered as “yes” then they were considered as low quality. Reviews that mainly focused on qualitative or mixed methods studies were appraised using the Joanna Briggs Institute (JBI) Critical Appraisal Checklist for Systematic Reviews [52]. This tool included eleven questions to guide the appraisal of quantitative and qualitative systematic reviews. We adopted a similar approach as the AMSTAR-2 and categorised our responses as “yes” or “no” to align with the scoring system. The quality appraisal tools were first piloted during team meetings with a sample of reviews (n = 5, 10%) to establish inter-rater reliability and then independently applied by three reviewers (SL, CMD, JF). Any inconsistencies between reviewers were discussed to achieve consensus.

### Data extraction

Data were independently extracted from the included reviews using a standardised Microsoft Excel form [53] by two reviewers (SL, CU). The extracted data were then cross-checked and verified by a third reviewer (CMD) to ensure accuracy and consistency. Any disagreements and inconsistencies were resolved through discussions. Key information extracted included:

- Study information (year of publication, number of articles reviewed, sample size).
- Information regarding the target audience (age of CYP, presenting mental health problems).
- Intervention-related information (type of digital support, predominant psychological underpinning theory).
- Outcome-related information (e.g., key findings in terms of clinical effectiveness, key findings at follow-up, and other findings such as cost, usability, suitability, adherence, retention, safety)

### Analysis and narrative summary

First, we described the characteristics of the identified studies, the target populations and information about the interventions. Key findings from the included reviews were then narratively summarised to address the research questions [45, 46].Unlike a narrative synthesis [54, 55], this narrative summary allowed a more descriptive and less structured way of presenting the findings, which allowed our community engagement and involvement activities to help shape the study. This process involved reading and organising the findings thematically and identifying patterns, similarities, and differences across the studies. When possible, we focused in the first instance on the highest quality and most recent reviews and supported this best evidence approach with evidence from less recent and lower quality reviews. Existing implementation science frameworks were used to guide the construction of themes [36, 39–41]. Due to the expected heterogeneity of reported interventions and outcomes, we did not aim to conduct a meta-analysis. The findings and emerging concepts were discussed and agreed during team meetings and community engagement activities.

### Community engagement and involvement

In line with best practice guidelines [56–58], this review benefited from the involvement of key stakeholders, including CYP. First, we partnered with a community youth organisation that offered online mental health services as a form of early intervention for CYP with a range of mental health problems. This collaboration provided a platform to facilitate shared learning. Practitioners and decision-makers were invited to scheduled meetings to co-create the research questions and offer views on the chosen methodology. Second, we collaborated with youth advisory groups that provide expert advice and guidance on research projects. We met with eight CYP between the ages of 12 and 18 who endorsed the relevance and value of the review. Third, we obtained feedback on the study plans and further insights on the relevance of the topic from a diverse group of parents and carers from a national charity. Together these stakeholders provided expert advice and lived experience insights on supported or self-managed DMHIs delivered at home, school, community and healthcare settings. Different stages of the synthesis and emerging concepts were also presented to the stakeholders for comments.

## Results

### Outcomes from community engagement and involvement

A total of seven community engagement meetings were held throughout the review process (45 to 90 min), of which three were in-person and four were online, providing feedback from eight young people, eleven parents/carers, four mental health practitioners and two service managers. In addition, there were six email discussions through opportunistic contact with key stakeholders (i.e., academics, commissioners) known to the research team. Some stakeholders contributed on multiple occasions. Notes were taken and summarised after each interaction, which guided the review process and informed the emerging themes and discussion points.

### Study selection

**Fig. 1:**
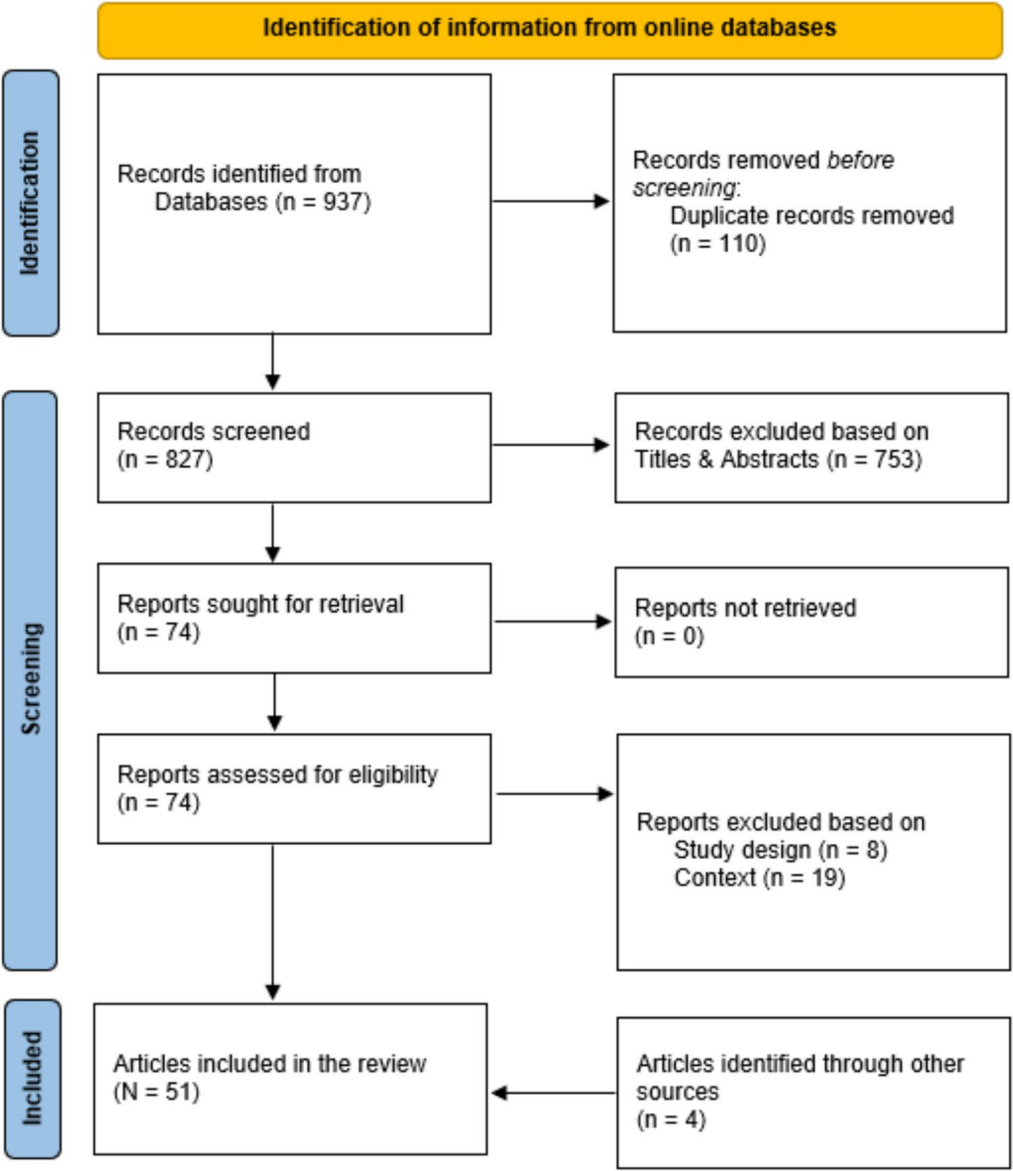
PRISMA Flow Chart documenting the article screening and selection process

A total of 937 records were retrieved after searching the six databases (Fig. 1). 827 records remained after duplicates were removed. After title and abstract screening, 74 publications were selected for full-text screening, of which 47 were eligible for inclusion in this umbrella review [28, 30, 59–103]. A further four articles were recommended by experts [104–107]. Most reviews were excluded based on contextual factors—not exploring the effectiveness of DMHIs on the mental health and wellbeing of CYP described in our eligibility criteria.

### Description of the reviewed articles

The included articles (N = 51) were published between 2000 and 2023 with just over half (n = 26, 51%) of the reviews conducted within the last five years. Most of the reviews (n = 28, 55%) included over 15 primary studies. Correspondingly, the total sample sizes captured by the reviews ranged from as low as two participants [60] to as high as 208,683 participants [96]. The identified reviews included a range of different interventions targeting CYP with various presenting mental health and wellbeing problems, which we describe in the following sections. Online Resource 3 also provides further details of the included articles.

### Quality of the reviewed studies

**Fig. 2:**
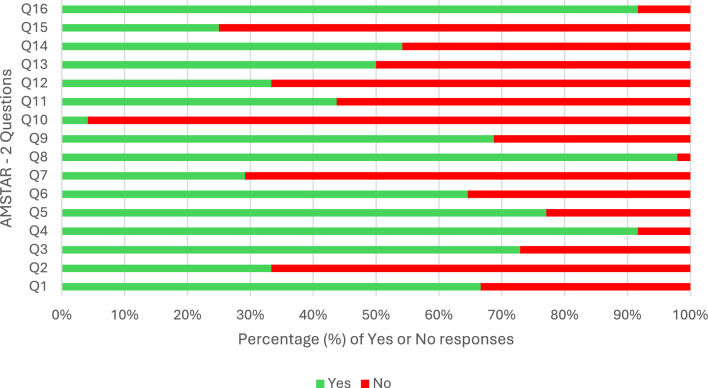
Summary of the AMSTAR-2 quality assessment. *AMSTAR, A MeaSurement Tool to Assess systematic Reviews

**Fig. 3:**
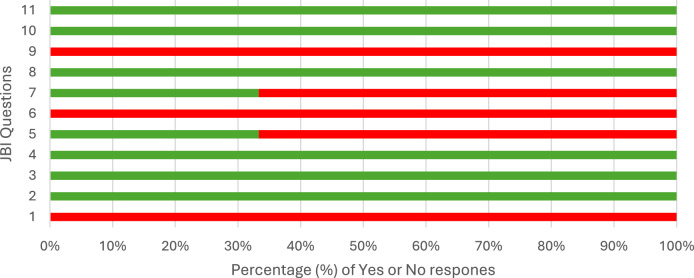
Summary of the Joanna Briggs Institute (JBI) Critical Appraisal Checklist for Systematic Reviews

Of the 51 articles, 48 reviews were assessed using AMSTAR-2. Six (13%) were judged as high quality based on the team’s confidence in the four key domains, while 31 reviews (65%) were judged as moderate quality and 11 (23%) were judged as low quality. Most of the reviews (32 out of 48, 67%) met at least eight of the 16 items on the AMSTAR-2. Most reviews had a detailed search strategy (44 out of 48, 92%), provided sufficient details of the reviewed papers (47 out of 48, 98%) and declared conflicts of interest (44 out of 48, 92%). However, most reviews (46 out of 48, 96%) did not report on the funding sources for the primary studies they reviewed (Fig. 2). Based on the four critical domains, 16 reviews (33%) registered a protocol before the start of the review, 44 reviews (92%) described a comprehensive search strategy, 14 reviews (29%) provided some detailed justification for excluding individual studies and 12 reviews (25%) adequately investigated publication bias. For the three reviews assessed using the JBI Critical Appraisal Checklist for Systematic Reviews and Research Syntheses, all were judged as moderate—one review met eight out of the eleven criteria and the remaining two met six out of the eleven (Fig. 3).

### Description of the reviewed samples

Based on the available evidence, DMHIs were being developed and delivered for CYP from as early as birth through early years (~ 5 years) [60–62, 65, 71, 73, 74]. For example, DMHIs were sometimes used to assess, monitor and record health and well-being outcomes, including behavioural concerns, from 0 to 5 years [e.g., 62]. Of the 51 reviews, 21 (41%) focused on CYP between the ages of 10 and 25 years. Although limited, one systematic review of ten primary studies also explored the use of DMHIs for CYP from low-income families [100].

DMHIs were designed for a range of internalising and externalising mental health symptoms/problems including low mood, depression, anxiety, stress, psychosis, conduct problems, eating disorders and obsessive compulsions, yet many reviews (n = 21, 41%) focused on anxiety and or depression only [30, 59, 69, 73, 76, 78, 79, 81, 83–86, 88, 92, 95, 97, 101, 104–107]. Some interventions also targeted factors associated with mental health problems such as loneliness, phobias, substance use, low self-esteem and insomnia [64, 68, 71, 82, 89, 103] or were offered to CYP with specific neurodevelopment disorders like attention-deficit/hyperactivity disorders (ADHD) and autism spectrum disorders (ASD) [28, 60, 72, 94]. The included reviews also explored DMHI’s usage among CYP experiencing varying levels of symptoms ranging from mild-moderate-severe, including general well-being issues, self-harm and suicidality [61, 62, 75, 87, 96, 100, 102].

### Description of the reviewed interventions

The DMHIs described in the reviewed articles adopted multiple formats and modalities. These included online live video calls [65, 66], text chats [68, 94] and mobile apps [67, 87, 89]. To increase engagement, several interventions incorporated games and immersive technology such as virtual reality and wearables (e.g., smart watches) [60, 80, 81, 83, 93].^.^ Some interventions were also linked to, or delivered through, social media or online networking platforms such as Facebook [e.g., 63, 82].

In 35 out of 51 articles, the identified DMHIs were predominantly underpinned by Cognitive Behaviour Therapy (CBT) or related theories [59, 61, 64, 65, 67–71, 73–75, 77–80, 84–86, 88, 92, 94–97, 99–103]. Our findings indicated that online CBT sometimes called iCBT (internet delivered CBT), dCBT (digital CBT), tCBT (technology-delivered CBT) and cCBT (computerised CBT) appear to be the most common online therapeutic support offered to CYP. These interventions were generally described as CBT-based structured psychological support delivered through online platforms such as apps or computer programs. Other less researched modalities included Solution Focused Therapy, Positive Psychology and Acceptance Commitment Therapy [28, 60, 63, 83, 87, 89, 91, 98]. In addition, some DMHIs were described as being delivered synchronously (i.e., in real-time with a therapist) or asynchronously (i.e. utilising self-help and self-management techniques) [e.g., 105, 106].

### Evidence of effectiveness

Of the 51 articles, 21 meta-analyses provided evidence suggesting that DMHIs had a positive effect on the mental health and well-being of CYP [28, 30, 59, 60, 69, 71, 73, 78, 82, 83, 85, 86, 91, 93, 95, 97, 99, 101, 102, 104]. Of these, two reviews were judged as high-quality evidence, 17 were judged as medium, and two were judged as low-quality (i.e., high risk of bias). The findings described DMHIs as effective in preventing, promoting and treating mental health symptoms such as depression, anxiety, distress, psychosis, suicidality and other externalising symptoms. For example, a review of 20 primary studies highlighted the efficacy of online support in preventing symptoms of major depressive disorders and suicide [77] while another review highlighted that online support significantly decreased the severity of obsessive–compulsive disorder (OCD) and related symptoms in CYP between 4 and 18 years old [74].

DMHIs were also generally described as being more effective in improving mental health outcomes than waitlist control groups when CYP were not receiving any care [e.g., 84, 95, 104, 106]. However, in some instances, online CBT was found to be as effective as offline CBT (i.e., in-person) and other active treatments in reducing anxiety and or depressive symptoms in CYP ages 10 to 25 years [95, 101]. In other studies, where online support was blended with offline activities, these also yielded promising outcomes for depressive symptoms in CYP 12 to 23 years [79]. Some evidence also suggested that specific components/features of the DMHIs such as facilitating social connections were active ingredients in making interventions effective [e.g., 83, 105].

Of the 51 articles, 10 articles described inconclusive or less promising evidence regarding positive mental health outcomes associated with DMHIs for CYP. For example, Pennant et al., [73] reported uncertainty around the effectiveness of cCBT in children 5–11 years despite positive effects on older children 12–25 years. Similarly, other authors reported that DMHIs significantly prevented or treated anxiety but not depression and vice versa, with even more uncertainty for other disorders [28, 30, 86, 90, 95]. There was also evidence describing uncertainty around the longer-term effects of DMHIs from as early as three months, since the positive effects of DMHIs were not maintained at follow-up [e.g., 83, 93, 96, 103].

### Evidence of acceptability

Of the 51 articles, 17 provided evidence on acceptability of DMHIs for CYP. Of these, two reviews were judged as high-quality evidence, 13 were judged as medium and two were judged as low-quality. The evidence suggested that DMHIs increased the acceptance of mental health interventions with the potential to overcome issues like social stigma during help-seeking [61, 63, 74, 75, 77, 90]. For example, one review of 20 studies reported that technologies were acceptable for preventing suicide prevention [77]. Although CYP generally had favourable responses to receiving DMHIs, there were some mixed findings on adherence and drop-out rates [61, 92, 98, 99]. For example, the lack of engagement was sometimes explained by usability issues related to the technology, while increased engagement was usually attributed to easier access, anonymity and gamification or visually appealing features of the DMHIs [60–63, 68, 72, 74, 77, 83, 88, 90, 100, 105, 106].

### Reduced cost and time investments

Of the 51 articles, 6 reviews reported additional benefits of DMHIs for CYP. Of these, one review was judged as high-quality evidence, four were judged as medium and one was judged as low-quality. In one article, DMHIs for CYP were viewed as useful for saving costs and travel time for CYP and their families when seeking mental health support [66]. Another review also highlighted that the cost savings were a significant motivator for engaging with online support over in-person visits [87]. Some authors also described continued usage of DMHIs as being related to the fact that CYP could access interactive therapeutic activities within their own space and on their own time [61, 68, 79]. However, the evidence base for the overall cost-effectiveness of DMHIs was not yet well established among the reviewed studies [e.g., 28].

### Access to suitable technology and connectivity

Of the 51 articles, only one review of medium quality explored DMHIs among socioeconomical and digitally marginalised CYP [100]. Yet it was noted that a key criterion for receiving support via DMHIs in many of the reviewed studies was having access to internet-accessible or smart devices [e.g., 69, 71, 74, 86, 88, 102, 107]. In the background literature, Piers et al. [100] discussed that some CYP may have issues accessing DMHIs due to underconnectivity to the internet, having to share devices with multiple individuals, having services disconnected due to nonpayment/low data or having to travel to schools or libraries to use specific technologies. Therefore, access to DMHIs for CYP went beyond owning a laptop or smartphone and highlighted a need to acknowledge other digital and socioeconomic factors that contribute to accessibility issues and ease of access.

### Ethical and safety concerns

Of the 51 articles, 6 reviews reported additional concerns of DMHIs for CYP. Of these four reviews were judged as medium-quality evidence and two were judged as low-quality. In addition to the accessibility issues described above, authors also reported that CYP’s smart devices alongside the DMHIs themselves, may need to be updated and developed over time to suit the needs of CYP [61, 66]. Hence, without the necessary upgrades and ongoing needs assessments, CYP may experience undue stress while connecting to, and attempting to remain engaged with DMHIs, which raises some ethical and safety concerns [e.g., 68]. Similarly, although technology allowed practitioners and parents/carers to monitor online group conversations more effectively, this raised concerns around the privacy and confidentiality of the therapeutic session [61, 63, 64, 88]. However, these steps were sometimes noted by parents/carers and practitioners as necessary to keep CYP safe while online [e.g., 61].

### Practitioner preparedness and training

Of the 51 articles, 10 reviews highlighted a need for ongoing support and development for practitioners who deliver DMHIs for CYP. Of these nine reviews were judged as moderate-quality evidence and one was judged as low-quality. The reviewed articles reported that implementing and integrating DMHIs into mental health services that were already facing high-pressured environments can be perceived as burdensome to mental health providers [62, 63, 66, 68, 87, 90]. Thus, feasibility studies and other strategies, such as staff training and service needs assessments, remained a priority across different studies [e.g., 61]. Several studies also highlighted a need for more effort and attention in tailoring DMHIs to meet the needs of CYP to help strengthen the therapeutic alliance between practitioners and CYP [74, 75, 101].

## Discussion

This umbrella review provides an updated overview of the available evidence on DMHIs for CYP, to help capture a comprehensive understanding of this rapidly evolving field. We summarised information from 51 literature reviews providing insights into the evidence base, intervention types and factors influencing the uptake, implementation and sustainability of effective DMHIs. This review further expands our knowledge of DMHIs’ usage and delivery for CYP from birth to 25 years experiencing, or at risk of, a wide range of mental health conditions, including anxiety and depression [31, 32, 35]. We identified popular digital innovations such as online video conferencing calls and mobile applications, as well as newer and emerging technological innovations such as virtual reality and immersive or augmented experiences that can be used when developing and implementing new DMHIs. We also confirmed the lack of diversity in target audiences and theoretical underpinnings as most DMHIs continue to focus on the principles of CBT. Nonetheless, the cumulative body of evidence provides promising findings on the clinical effectiveness of DMHIs after the intervention period, but with limited evidence for cost-effectiveness and longer-term outcomes [23, 84, 86]. Our summary of the key findings also highlights the importance of acceptability, accessibility, ethical and online safety, cost and time investment of CYP and their family, as well as practitioner preparedness and training. These factors add to findings from similar reviews [33–35] to create favourable conditions for the feasibility, implementation and ongoing usage of DMHIs to benefit CYP. The current study also fills the gaps where previous umbrella reviews have mainly focused on the effectiveness of DMHIs for specific populations, disorders and types of DMHIs.

One significant contribution to knowledge is that the above findings provide further insights that can be used to inform the upgrade and refinement of existing implementation science frameworks [36, 39–41]. For example, this review confirms that factors such as online safety and practitioner training remain critical for the success and sustainability of DMHIs for CYP. Although studies have begun to identify barriers and facilitators related to acceptability and effectiveness, this review confirms that accessibility, cost, safety and practitioner training are important factors to consider if DMHIs are to be sustainable beyond the implementation phase [42, 108]. Therefore, our findings can be used to inform future decision-making related to policies, practice and research [19, 20, 109]. Addressing these factors would require appropriate co-production so end users can be involved in the design, development and eventual implementation of DMHIs [110]. Adopting this approach will ensure DMHIs are acceptable and feasible for CYP and those who support them [24, 111, 112]. This can then positively impact ongoing usage and reduce the risk of drop-out from online services.

Knowledge of factors contributing to health inequalities and those factors that widen the access to treatment gaps for some groups of CYP also remains a key priority [100, 113]. Alongside other key recommendations [32], our findings contribute to the advancements and improvements in this area, by suggesting additional benefits of DMHIs and accessibility issues that should be considered when making DMHIs culturally responsive. Piers et al. [100] suggested that DMHIs can benefit CYP from low-resource communities. Therefore, the findings from this review further strengthen the call for more research and policies to ensure DMHIs are designed to support all CYP from different backgrounds and in different settings [114–116]. It was noted that none of the identified reviews targeted other marginalised groups, such as ethnic and gender minority groups, which is consistent with national and international research priority areas. However, the large number of identified reviews pointing towards the clinical effectiveness of DMHIs could be an indication of potential success if DMHIs are to be adopted for various marginalised groups [19].

Moreover, online safety and practitioner preparedness and training are an ongoing debate [117]. In particular, balancing CYP’s privacy with online monitoring is a complex ethical and practical challenge. On one hand, privacy is crucial for fostering trust and encouraging honest engagement with mental health support. In contrast, unrestricted privacy can pose risks, particularly for vulnerable users who may require external intervention in times of crisis. To address this, the voices of CYP alongside clinicians’ concerns should be taken into account during the design and development stages, and both parties should be supported to use technology to enhance the online therapeutic experience. Based on the available evidence, ongoing research is also needed to identify the long-term benefits of online support, cost-effectiveness and new ways to keep CYP engaged while receiving mental health support. Once achieved, DMHI developers and researchers could continue to develop and scale up effective DMHIs to reduce the high prevalence of CYP’s mental health problems globally [1–5].

There are also gaps in the existing evidence base that could be further investigated. For example, more research targeting younger CYP under 10 years is needed since recent studies suggest that this age group have been significantly impacted by the recent COVID-19 pandemic [11]. Similarly, due to the rapid advancements and dynamic nature of technology, developers and researchers can continue to explore non-CBT-based interventions, like the use of digitised creative arts therapies to meet the needs of CYP who may not benefit from CBT [118]. It is possible that these investigations can help us understand why some DMHIs work under some circumstances and others may not. Another priority area needing further investigation is adherence and dropout rates when CYP engage with DMHIs. Although there is some evidence suggesting high levels of engagement [e.g., 24], there is also evidence highlighting low programme completion [e.g., 119]. This mixed finding suggests further investigations into dropout reasons that go beyond technical issues. For example, are CYP dropping out from online support because they are feeling better or are they too unwell to continue engaging?

### Strengths and limitations

Although this umbrella review provides a broad overview and quality assessment of the available evidence on DMHIs for CYP, some limitations to our approach and methodological decisions must be acknowledged. We utilised a robust methodological approach using a comprehensive search strategy in six academic databases to identify relevant articles, however, some studies could have been missed and our findings are still limited by the available evidence from the reviews that met our inclusion criteria. Nonetheless, a large number of systematic reviews (N = 51) were identified which provides a broad and comprehensive scope of the evidence. Further, by relying on the evidence and conclusions reported by the authors in the primary reviews we were limited to a higher-level descriptive analysis and narrative summary. Any further insights (e.g., subgroup analyses) would require a secondary analysis of the primary studies included in the reviewed reviews, which was beyond the scope of this study. We also acknowledge that the inclusion criteria applied in this review may have resulted in the overlap of primary studies between the reviews. While assessing primary study overlap can help quantify redundancy and avoid overrepresentation of findings, our primary aim was to synthesise overarching conclusions rather than conduct a meta-analysis [45, 46]. It is also important to note that reviews of different levels of quality were pooled, each with its own limitations as described in the AMSTAR-2 and JBI quality appraisal process. Similarly, our review only focused on literature published in English that discussed the effectiveness of DMHIs. We acknowledge publication bias in that many programmes that are not effective and studies not published in English could still generate learnings for uptake, implementation and sustainability of DMHIs for CYP. Furthermore, many of the included reviews limited their search language to English or did not search for gray literature (e.g., non-academic publications), potentially increasing publication bias. Lastly, the included reviews generally were of moderate quality which must be taken into consideration when interpreting the findings of our study.

To ensure the creditability and reliability of our review, we followed a predetermined protocol and involved at least two reviewers during screening and study selection, quality appraisal, data extraction and analysis process to increase rigour. Another significant strength of this umbrella review was the input from CYP, parents/carers, practitioners and decision-makers during the community engagement and involvement activities. Their input guided the research questions and study process, thereby making the research aims, methods and findings align with the needs of key stakeholders.

## Conclusions

To the best of our knowledge, this umbrella review is the largest and most up-to-date study to confirm the promise of DMHIs in improving not just clinical and psychosocial outcomes for CYP, but also in increasing access to effective mental health support and acceptable forms of interventions. The significance of this umbrella review is two-fold. First, the themes highlighted provide opportunities for further research into the integration and delivery of DMHIs for CYP. Second, as a rapidly advancing mode of intervention delivery, this review provides an updated understanding of the potential factors that could enhance engagement and potential effectiveness of DMHIs for CYP.

## Supplementary Information

Below is the link to the electronic supplementary material.

Supplementary file1 (DOCX 425 KB)

## Data Availability

No datasets were generated or analysed during the current study.
