# Supplementary material for: Updates on digital mental health interventions for children and young people: systematic overview of reviews
Source: Eur Child Adolesc Psychiatry. 2025 Apr 25;34(10):2961–74. doi: 10.1007/s00787-025-02722-9 (PMC12592321; doi:10.1007/s00787-025-02722-9)
Supplement: Supplementary file 1 — Supplementary file1 (DOCX 425 KB) [file 787_2025_2722_MOESM1_ESM.docx]

# Online Resource 1. PRIOR Checklist

| **Section**  Topic | **#** | **Item** | **Location reported** |
| --- | --- | --- | --- |
| **TITLE** | | |  |
| Title | 1 | Identify the report as an overview of reviews. | Page 1 |
| **ABSTRACT** | | |  |
| Abstract | 2 | Provide a comprehensive and accurate summary of the purpose, methods, and results of the overview of reviews. | Page 2 |
| **INTRODUCTION** | | |  |
| Rationale | 3 | Describe the rationale for conducting the overview of reviews in the context of existing knowledge. | Page 3 |
| Objectives | 4 | Provide an explicit statement of the objective(s) or question(s) addressed by the overview of reviews. | Pages 5,6 |
| **METHODS** | | |  |
| Eligibility criteria | 5a | Specify the inclusion and exclusion criteria for the overview of reviews. If supplemental primary studies were included, this should be stated, with a rationale. | Page 7 |
|  | 5b | Specify the definition of ‘systematic review’ as used in the inclusion criteria for the overview of reviews. | Page 7 |
| Information sources | 6 | Specify all databases, registers, websites, organizations, reference lists, and other sources searched or consulted to identify systematic reviews and supplemental primary studies (if included).  Specify the date when each source was last searched or consulted. | Page 6 |
| Search strategy | 7 | Present the full search strategies for all databases, registers and websites, such that they could be reproduced. Describe any search filters and limits applied. | Page 6,7 |
| Selection process | 8a | Describe the methods used to decide whether a systematic review or supplemental primary study (if included) met the inclusion criteria of the overview of reviews. | Page 6 |
|  | 8b | Describe how overlap in the populations, interventions, comparators, and/or outcomes of systematic reviews was identified and managed during study selection. | Page 7 |
| Data collection process | 9a | Describe the methods used to collect data from reports. | Page 8 |
|  | 9b | If applicable, describe the methods used to identify and manage primary study overlap at the level  of the comparison and outcome during data collection. For each outcome, specify the method used to illustrate and/or quantify the degree of primary study overlap across systematic reviews. | N/A |
|  | 9c | If applicable, specify the methods used to manage discrepant data across systematic reviews during data collection. | N/A |
| Data items | 10 | List and define all variables and outcomes for which data were sought. Describe any assumptions made and/or measures taken to identify and clarify missing or unclear information. | N/A |
| Risk of bias assessment | 11a | Describe the methods used to *assess* risk of bias or methodological quality of the included systematic reviews. | Page 7,8 |
|  | 11b | Describe the methods used to *collect* data on (from the systematic reviews) and/or *assess* the risk of bias of the primary studies included in the systematic reviews. Provide a justification for instances where flawed, incomplete, or missing assessments are identified but not re-assessed. | N/A |
|  | 11c | Describe the methods used to *assess* the risk of bias of supplemental primary studies (if included). | N/A |
| Synthesis methods | 12a | Describe the methods used to summarize or synthesize results and provide a rationale for the choice(s). | Page 8,9 |
|  | 12b | Describe any methods used to explore possible causes of heterogeneity among results. | N/A |
|  | 12c | Describe any sensitivity analyses conducted to assess the robustness of the synthesized results. | N/A |
| Reporting bias assessment | 13 | Describe the methods used to *collect* data on (from the systematic reviews) and/or *assess* the risk of bias due to missing results in a summary or synthesis (arising from reporting biases at the levels of the systematic reviews, primary studies, and supplemental primary studies, if included). | N/A |
| Certainty assessment | 14 | Describe the methods used to *collect* data on (from the systematic reviews) and/or *assess* certainty (or confidence) in the body of evidence for an outcome. | N/A |
| **RESULTS** | | |  |
| Systematic review and supplemental primary study selection | 15a | Describe the results of the search and selection process, including the number of records screened, assessed for eligibility, and included in the overview of reviews, ideally with a flow diagram. | Page 9 |
|  | 15b | Provide a list of studies that might appear to meet the inclusion criteria, but were excluded, with the main reason for exclusion. | N/A |

# Online Resource 2.

# MEDLINE search strategy

| TI (child* OR youth* OR young OR adolesce* OR teenage* OR pre*teen* OR kid* OR girl* OR boy* OR student* OR infan* OR toddler* OR kid*) | “Mental health”/ OR well*being OR Depression/ OR Emotion*/ OR Stress OR anxiety OR Psychological/ OR "mental wellbeing" ("Mental ADJ3 Health" or Depression or Emotion* or "Psychological ADJ3 stress" or "Psychotic ADJ3 Disorder*" or Mental Disorder or "mental ADJ3 difficult*" or "mental health challenge*" or "mental health problem*" or "mental challenge*" or "mental well?being" or "mental health well?being" or anxiety* or depress* or "low?mood" or worry or psychiatr* or psycho* or sad* or lonl* or aggressi* or "behavio?r ADJ3 problem*" (MM "Behavioral and Mental Disorders") OR (MM "MentalDisorders") OR (MM "Depression") OR (MM "Anxiety") OR(MM "Emotional Lability") (MM "Telepsychiatry") OR (MM "Telemedicine") OR (MM"Telehealth") | TI “computer assisted therap*” or internet or computer* or“mobile devices” or website* or online or app* or smartphone* or "mobile phone*" or digital* or technolog*or computer* or mhealth* or m#health or “mobile health” or e#health or "electronic health" or phone or online or web#based or VR or “virtual reality” N2 (treatment* or intervention* or therap* or training or support* or help* or assistan*) or web N2 (treatment* or intervention* or therap* or training or support* or help* or assistan*) or avatar* or chatbot* N2 (treatment* or intervention* or therap* or training or support* or help* or assistan*) or telemedicine or “Remote consultation*” or telepsychiatry or telehealth or e-medicine or "computer-assisted diagnosis" or "virtual medicine" | AB ( (systematic or structured or evidence or intergtative or literature or narrative or scoping) N2 (review or overview or look or examination or update* or summary ormeta‐analys* or meta analys* or metaanalys* or metasynth* or meta‐synth* or metasynth*) ) OR TI ( (systematic or structured or evidence or intergtative or literature or narrative or scoping) N2 (review or overview or look or examination or update* or summary or meta‐analys* or meta analys* or metaanalys* or meta synth* or meta‐synth* or metasynth*) ) | limit to (human and english language and ("reviews (maximizes sensitivity)" or "reviews (maximizes specificity)" or "reviews (best balance of sensitivity and specificity)") and ("literature review" or "systematic review" or “meta analysis” or “metasynthesis”) and (childhood <birth to age 12 yrs> or 200 adolescence <age 13 to 17 yrs>) and english and human) |
| --- | --- | --- | --- | --- |

**CINAHL search strategy**

| TI ( child* OR youth* OR young OR adolesce* OR teenage* OR pre*teen* OR kid* OR girl* OR boy* OR student* OR infan* OR toddler* OR kid* ) OR AB ( child*OR youth* OR young OR adolesce* OR teenage* OR pre*teen* OR kid* OR girl* OR boy* OR student* OR infan* OR toddler* OR kid* ) (MH "Child, Preschool") OR (MM "Infant, Newborn") OR(MM "Infant") OR (MM "Child" | TI ( “Mental N3 Health” or Depression or Emotion* or“Psychological N3 stress” or “Psychotic N3 Disorder*” or Mental Disorder or "mental N3 difficult*" or "mental health challenge*" or "mental health problem*" or "mental challenge*" or "mental well#being" or "mental health well#being" or anxiety* or depress* or “low?mood” or worry or psychiatr* or psycho* or sad* or lonl* or aggressi*or “behavio#r N3 problem*” or "Emotional Lability" or "Emotional Instability") OR AB ( “Mental N3 Health” or Depression or Emotion* or “Psychological N3 stress” or “Psychotic N3 Disorder*” or Mental Disorder or "mental N3difficult*" or "mental health challenge*" or "mental healthproblem*" or "mental challenge*" or "mental well#being" or "mental health well#being" or anxiety* or depress* or “low?mood” or worry or psychiatr* or psycho* or sad* or lonl* or aggressi* or “behavio#r N3 problem*” or "Emotional Lability" or "Emotional Instability") (MM "Behavioral and Mental Disorders") OR (MM "MentalDisorders") OR (MM "Depression") OR (MM "Anxiety") OR (MM "Emotional Lability") (MM "Psychotherapy") OR (MM "Mental Health Services") | TI ( “computer assisted therap*” or internet or computer*or “mobile devices” or website* or online or app* or smartphone* or "mobile phone*" or digital* or technolog*or computer* or mhealth* or m#health or “mobile health” ore#health or "electronic health" or phone or online or web#based or VR or “virtual reality” N2 (treatment* or intervention* or therap* or training or support* or help* or assistan*) or web N2 (treatment* or intervention* or therap* or training or support* or help* or assistan*) or avatar* or chatbot* N2 (treatment* or intervention* or therap* or training or support* or help* or assistan*) or telemedicine or “Remote consultation*” or telepsychiatry or telehealth or e-medicine or "computer-assisted diagnosis" or "virtual medicine") (MM "Internet-Based Intervention") (MM "Telepsychiatry") OR (MM "Telemedicine") OR (MM"Telehealth") (MM "Diagnosis, Computer Assisted") OR (MM "Therapy,Computer Assisted") | Limiters - English Language; Human; Publication Type: Meta Analysis, Meta Synthesis, Review, Systematic Review;  Age Groups: All Child  Expanders - Apply equivalent subjects |
| --- | --- | --- | --- |

**PsycInfo search strategy**


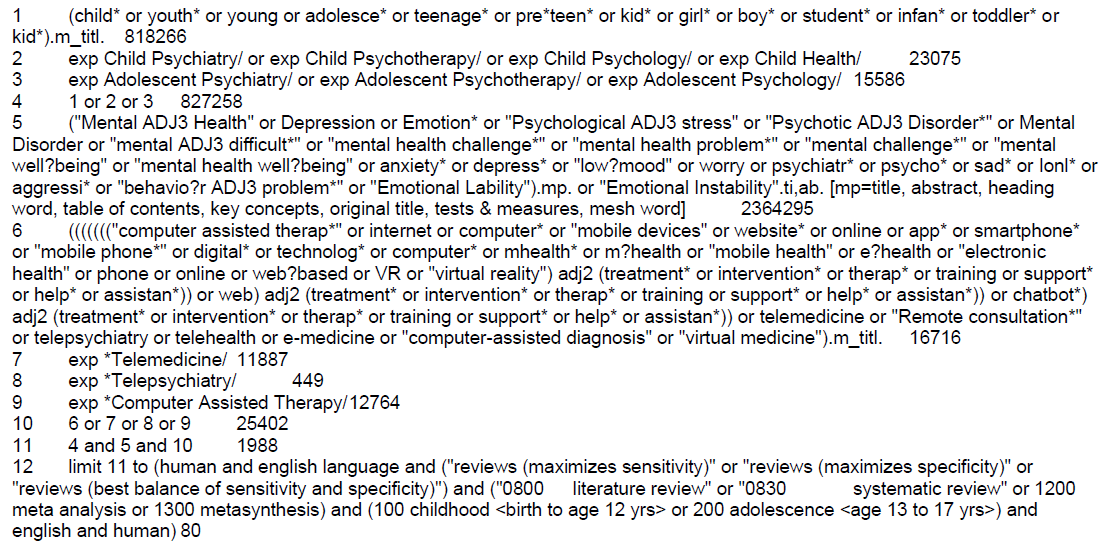


**Scopus search strategy**


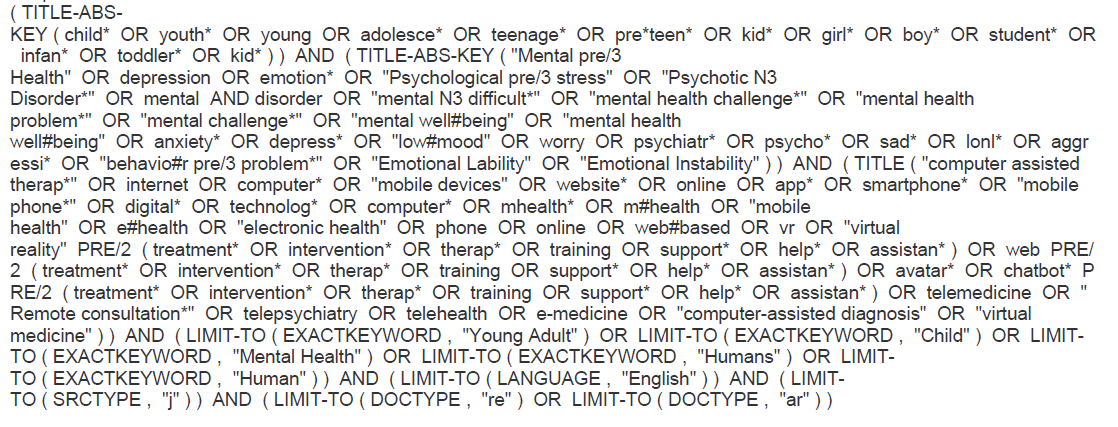


**AMED search strategy**


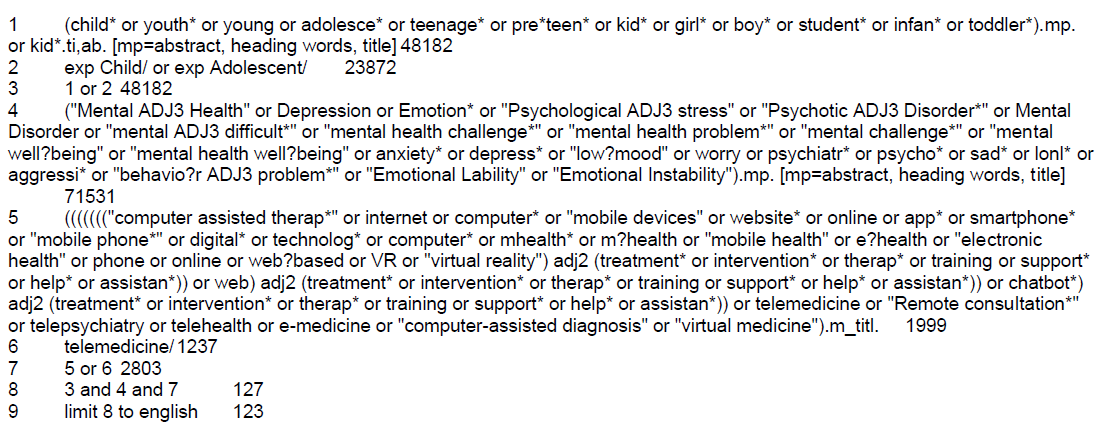


# Online Resource 3. Characteristics of the reviewed studies

|  | **First Author, Year of Publication** | **No. Of Studies Included** | **Sample Size** | **Age of CYP (years)** | **Presenting Disorder(s)** | **Predominant Psychological Underpinning** | **Predominant Technology Described** | **Key Findings** |
| --- | --- | --- | --- | --- | --- | --- | --- | --- |
| 1 | Noh, 2023 | 22 | Range 21 to 369 | 13 to 25 | Depression (mild to severe) | CBT | Internet/online Mobile apps | Online interventions, specifically iCBT, are recommended for adolescents and young adults with depression, but further studies are required to confirm their long-term effects. |
| 2 | Howes, 2023 | 6 | N - 606, Range 44 to 187 | 10 to 25 | Depression, Anxiety | CBT | Online/Offline | The effectiveness of online CBT in reducing anxiety and depressive symptoms among young individuals, including those in the initial stages of symptom manifestation, seems comparable to traditional, face-to-face CBT. |
| 3 | Piers, 2023 | 10 | Range 9 to 313 | Up to 25, Mean 16.89 | General mental health | CBT | Mobile apps Internet/online Offline games Phone calls/ SMS | There is moderate evidence suggesting that digitally delivered interventions can be effective in improving mental health outcomes among socioeconomically and digitally marginalised youth, but more high-quality research is needed in order to determine whether online support can fully bridge the so-called ‘digital divide’. |
| 4 | Wright, 2023 | 27 | N - 13,857; Range 22 to 1841 | 10.9 to 17.9, Mean 16.65 | Anxiety, depressive symptoms, externalizing and internalizing symptoms, protective factors, stress, and well-being | CBT, mindfulness, growth mindset theory, social-emotional learning theory | Digital tools | Regarding promoting well-being, relieving anxiety and enhancing protective factors, digital tools were found to be promising. |
| 5 | Noh, 2022 | 19 | Not clear | 10 to 24 | Depression, anxiety, stress | CBT | Online interventions | The meta-analysis showed that online interventions significantly prevent an increase in depression score but not in stress and anxiety scores. |
| 6 | Ramshaw, 2023 | 27 | Range 2 to 1645 | 4 to 18 | General mental health | CBT | Online platforms, websites, apps/games, video-related technology, SMS/phone calls | Technology efficacy in youth mental health services relies on its usability, efficiency, and capability to effectively engage young individuals. |
| 7 | Babbage, 2022 | 12 | N - 1460, Range 23 to 240 | 11 to 24, Mean 15.4 | Anxiety, depression, stress, low mood, social functioning difficulties | CBT | Mobile-phones Internet/online and offline computer programmes | This review emphasizes the importance of expanding self-help interventions to provide stronger support for vulnerable young people facing challenges with their mental well-being. |
| 8 | Cervin, 2022 | 9 | Not clear | Not clear | Anxiety Disorders | CBT | Technology-delivered inteventions | In pediatric anxiety disorders, tCBT moderately contributes to remission and improved clinician-rated functioning. It has a small impact on caregiver-reported anxiety, with no statistically significant effect on youth-reported anxiety. |
| 9 | Wickersham, 2022 | 16 | N – 4012, Range 20 to 1477 | 11 to 19 | Depression, Anxiety | CBT | Any digital, computerized, or web-based program | This study emphasises the effectiveness of cCBT for the treatment of anxiety and depression in adolescents. |
| 10 | Ivlev, 2022 | 12 | N - 1575 | 12 to 18 | Depression | CBT | Computer- or web-based programs administered via computers, iPads, or cellphone and computer video game | A limited body of evidence suggests that dCBT programs might outperform control interventions for reducing depressive symptoms immediately post-intervention, but not at a longer follow-up. The safety of dCBT programs for adolescents with depression is understudied. |
| 11 | Zhou, 2022 | 14 | N - 11616 | 10 to 19 | Self esteem, depression, stress, loneliness | Not clear | Online social support (Facebook, Instagram and Twitter) | Online social support might be beneficial for adolescents' mental health, especially self-esteem, although the causal relationship requires longitudinal studies to confirm, and the underlying mechanisms need further investigation. |
| 12 | Stewart, 2022 | 39 | Range 7 to 149, 326 | Birth - 24 | General mental health, suicide | Not clear | Internet- or mobile-based technology | eHealth tools that assess and track health in CYP have the potential to enhance health service delivery; however, a strong evidence base validating the clinical utility, efficacy, and safety of tools is lacking. |
| 13 | Reynard, 2022 | 39 | Range 2 to 1645 | 5 to 17 | Anxiety, ASD, Post-traumatic stress disorder, anorexia nervosa | Social and cognitive skills training | Digital games, biofeedback, Virtual and augmented reality | There is cause to be optimistic about digital interventions supporting the difficulties that youth experience in emotion regulation, but more work is needed in this area. |
| 14 | Zhou, 2021 | 45 | N – 13,291, Range 61 to 1767 | Range 14.8 to 26.7 | Depression, Anxiety,  Stress | CBT, Mindfulness,  Acceptance commitment therapy | Web/mobile app  AI/Online chats | Online interventions for mental health were shown to be effective in addressing various mental health conditions in young individuals. |
| 15 | Leech, 2021 | 11 | N- 1706 | Range 10 to 35 | Distress, stress, anxiety, general mental health, depression | Not clear | Apps/Game-like features | Smartphone apps hold promise as a stand-alone self-management tool in mental health service delivery. Further controlled trials with follow-up data are needed to confirm these findings as well as determine treatment engagement and effectiveness across diverse groups of participants. |
| 16 | Buttazzoni, 2021 | 12 | Range 20 to 120 | Youth | Internalising disorders - anxiety, depression or stress | CBT, emotional self-awareness, positive psychology, social interaction | Smartphone-based interventions | The overall pooled effect from the meta-analysis showed small but significant effects for interventions in reducing the symptoms of internalizing disorders. |
| 17 | Dewa, 2021 | 42 | N – 23,319 | 14 to 24 | Depression, anxiety | Social Support Theories | Smartphone apps, virtual reality packages, internet-based treatment and chat rooms, telepsychiatry, video gaming | Digital-Quality Social Connection is crucial for youth depression and anxiety outcomes, future research should focus on improving Quality Social Connection in digital interventions. |
| 18 | Kothgassner, 2021 | 4 | N - 100 | 8 to 16 | Anxiety | Developmental and Clinical Psychology | Virtual Reality | Participants show clinical improvements regarding anxiety symptoms after VRET. |
| 19 | Halldorsson, 2021 | 19 | Range 2 to 187 | 7 to 19 | Anxiety, depression, and phobias | CBT | Applied games and Virtual Reality | Before applied games and virtual reality are used as treatments for CYPs with mental health, it is important that more research is carried out to determine their effectiveness. |
| 20 | Forte, 2021 | 20 | Range 9 to 63,252 | 10 to 25 | Suicide, Depression, Anxiety | CBT | Telemedicine (web based), mobile applications, and language detection software | Emerging technologies were accepted and tolerated for preventing adolescent suicide. |
| 21 | Christ, 2020 | 24 | Range 19 to 257 | 13.3 to 24.4 | Anxiety and depressive disorders | CBT | Computer/Online | Compared with passive controls, cCBT is more beneficial for post treatment and depressive symptoms reduction in adolescents and young adults. |
| 22 | Punukollu, 2019 | 4 | Not clear | 10 to 29, Mean 18.1 | Depression, anxiety and suicide | CBT | Mobile apps, Automated phone calls | Mental health apps designed for young individuals can be vital tools for assessment, management, and treatment, improving service accessibility and aiding in prevention and self-help. Despite their potential, more studies are required to further understand their effectiveness. |
| 23 | Rasing, 2019 | 33 | Range 9 to 1239 | 12 to 23 | Depression, anxiety | CBT | Computer-based, internet-based | Computerised approaches seem promising for treatment of depression in youths. |
| 24 | Grist, 2019 | 34 | N – 3113, | 6 to 18 | Depression, anxiety | CBT | Computers, CD-ROM, internet, smartphones, virtual reality. | CBT based interventions are effective especially where access to traditional psychotherapies is limited or delayed. |
| 25 | Garrido, 2019 | 56 | Range 2 to 8207 | 10 to 25 | Depression, anxiety | CBT, positive psychology, ACT, motivational interviewing, Moderated Online Social Therapy, Spiritual health, Theory of planned behaviour | Online, phone, games, avatar-based | Digital interventions work better than no intervention to improve depression in young people when results of different studies are pooled together. However, these interventions may only be of clinical significance when use is highly supervised. |
| 26 | McCashin, 2019 | 14 | Not clear | 6 to 18 | Low mood, anxiety, trauma or self-harm, and physical difficulties | CBT | Web-based, mobile apps, games | Overall, young people’s experiences with tech-assisted CBT were mostly positive. One theme emphasized that young people found online support particularly helpful and increased enjoyment |
| 27 | Babiano-Espinosa, 2019 | 6 | Not clear | 4 to 18 | Obsessive-compulsive disorder (OCD) | CBT | Internet technology, educative texts, films | Evidence regarding acceptability, feasibility, and efficacy of online support for paediatric OCD is promising but this needs to be confirmed and refined in further research. |
| 28 | Barnes, 2018 | 5 | Not clear | Adolescents | Anxiety | CBT, attention bias modification | Video games | Early findings suggest that therapeutic games have potential in helping to engage adolescents with anxiety and lead to clinically measurable reductions in symptoms. |
| 29 | Dubad, 2018 | 25 | Range 6 to 108,996 | 10.95 to 23.7 | Depression, high-functioning autism/Asperger’s disorder, substance or alcohol | Not clear | Mobile mood-monitoring applications | Although mood-monitoring apps show promise for youth, the evidence is limited due to a lack of high-quality studies. Future research should focus on their impact on self-regulation, clinical outcomes, and youth engagement with mental health services, while also investigating potential negative effects suggested by other studies. |
| 30 | Ridout, 2018 | 9 | Not clear | Up to 25 | Mental health issues (depression and psychosis), mood disorder, mental health literacy, social support, and general well-being | Social Therapy Theory | Moderated online social therapy (MOST) model, Facebook, video games and mobile apps | Social networking sites-based interventions were found to be very valuable by young people. |
| 31 | Hollis, 2017 | 30 | Not clear | Up to 25 | Attention deficit disorders, autism, anxiety, depression, psychosis, eating disorders, post-traumatic stress disorders | CBT, Solution focused therapy | Digital tools | There is some support for the clinical benefit of online interventions, particularly cCBT, for depression and anxiety in adolescents and young adults but more research is needed to draw definitive conclusions. |
| 32 | MacDonell, 2017 | 13 | Not clear | 6 to 16 | Autism, Self-esteem, Anxiety, Depression | CBT | CD/Text/Chat | Technology-based interventions focused on youth and families led to some reductions in depressive/anxiety symptoms. |
| 33 | Grist, 2017 | 24 | Not clear | 9 to 30 | Depression, stress, anxiety, body image, self-esteem, and obsessive compulsive disorder | CBT and motivational interviewing | Apps | Two small randomized trials and one case study failed to demonstrate a significant effect of three apps on intended mental health outcomes. |
| 34 | Podina, 2016 | 6 | N - 340 | Up to 18 | Anxiety | CBT | Computer/internet | The results indicated that eCBT was as effective as standard CBT and more effective than waitlist (g = 1.410) in reducing anxiety symptoms. |
| 35 | Stasiak, 2016 | 12 | Range 19 to 1477 | 7 to 18 | Depression, anxiety, | CBT, Cognitive Bias Modification, Attention Bias Modification | Computer-based, online, CD-ROM | Computerized treatments for youth with depression and anxiety are effective, enhancing the availability of psychological therapies for children. |
| 36 | Vigerland, 2016 | 25 | N ~ 1882 | 3 to 21 | Social anxiety disorder, insomnia, depression, mixed anxiety disorders, asthma, chronic fatigue, chronic and recurrent pain, conduct problems, depressed mood, encopresis, functional gastrointestinal disorders, obsessive compulsive disorder and overweight | CBT | Internet-based platforms | CBT for psychiatric and somatic conditions in CYP can be successfully adapted to an internet-delivered format. |
| 37 | Struthers, 2015 | 24 | Not clear | 7 to 25 | Eating disorders, depression, anxiety and obsessive compulsive disorder | Not clear | Not clear | e-mental health seems to be a suitable intervention option for children, adolescents, and young adults, as well as their parents and healthcare providers. |
| 38 | Pennant, 2015 | 27 | Not clear | 5 to 25 | Anxiety, depression | CBT | Internet-downloadable software, CD-ROMs or smartphone applications. | There is evidence that cCBT is effective for treating and preventing anxiety and depression in young people, both in clinical and general settings. However, further research is necessary to broaden its utilization and validate its advantages in children. |
| 39 | Clarke, 2015 | 28 | Not clear | 12 to 25 | Stress, depression, anxiety | CBT | Web-based, social network electronic game, mobile phone | There is significant positive effect of cCBT in promoting wellbeing and improving mental health outcomes of adolescents. |
| 40 | Rooksby, 2015 | 6 | Range 9-941 | 7 to 16 | Anxiety | CBT | Computer-assisted programme | The findings support utilizing online delivery to increase the availability of evidence-based therapy. |
| 41 | Ebert, 2015 | 13 | N – 796 | 6 to 25 | Anxiety, depression | CBT | Computer, internet, mobile interventions | Results provide evidence for the efficacy of cCBT in the treatment of anxiety and depressive symptoms in youth. |
| 42 | Rice, 2014 | 15 | Not clear | Young people | Depression | CBT | Online and social networking sites | Internet-based interventions adopting a comprehensive cognitive-behavioural show potentials in alleviating symptoms of depression in young individuals. |
| 43 | Ye, 2014 | 7 | N – 569 | 7 to 25 | Anxiety and/or depression | Not clear | Internet-based | Online interventions effectively reduced anxiety symptoms and increased remission rates, but did not effectively decrease the severity of depression symptoms. |
| 44 | Boydell, 2014 | 126 | Not clear | Children and youth | General mental health | CBT and family-based interventions, ACT | Videoconferencing, telephone and mobile phone applications and Internet-based applications such as email, websites and CD-ROMs | Adopting technology interventions enhances the delivery of mental health services for CYP. |
| 45 | Seko, 2014 | 17 | Not clear | 13 to 24 | Depression, anxiety, including substance and alcohol use disorders | CBT | Mobile phones (SMS, apps, or multimedia messaging system) | The flexibility, interactivity, and spontaneous nature of mobile communications are considered advantageous in encouraging persistent and continual access to care outside of clinical settings, but ethical issues should also be considered. |
| 46 | Reyes-Portillo, 2014 | 25 | Not clear | 5 to 25 | Depression, anxiety, and suicide | CBT | Internet, mobile app | More evidence is needed to support the effectiveness of Web-based interventions for youth depression and anxiety. |
| 47 | Adelman, 2014 | 40 | N – 2648 | Young adults and Children | Anxiety | CBT | Computer-based, | cCBT represents an efficacious intervention for the treatment of anxiety disorders and may circumvent barriers to accessing traditional CBT treatments. Further research is needed to examine the effectiveness of cCBT in real-world settings. |
| 48 | Slone, 2012 | 35 | Not clear | 4 to 18 | Smoking/alcohol/drug use, eating disorders or disordered eating, and emotional distress | CBT | Videoconferencing, Internet and telephone | While there is evidence to support use of technology interventions in improving mental health outcomes of children and adolescents, there is need to further explore telepsychology within professional psychology. |
| 49 | Siemer, 2011 | 32 | Not clear | 9 to 18 | Depression, anxiety, substance abuse, eating disorders, and general mental health | CBT | Web-based | There is a growing body of literature supporting effectiveness of internet mental health interventions, with small to moderate effect sizes observed in this review. These may be acceptable, given the potential for low cost and straightforward distribution. |
| 50 | Richardson, 2010 | 10 | Range 2 to 189 | 7 to 25 | Depression and anxiety | CBT | Online | Studies consistently show that computer-based cognitive-behavioural therapy (cCBT) is effective in reducing clinical symptoms and improving well-being for children. While treatment satisfaction is high, dropout rates are a concern, emphasising the need for further research. |
| 51 | PesÃmaa, 2004 | 27 | Not clear | 8 to 14 | Depression, anorexia nervosa, disruptive behaviour problems | Not clear | Videoconferencing | Child telepsychiatry studies highlight benefits of videoconferencing such as improved accessibility, education, and potential savings. |

# Online Resource 4. Quality assessment of the reviewed studies

**Assessing the Methodological Quality of Systematic Reviews (AMSTAR)**

| First Author (year) | Q1 | **Q2** | Q3 | **Q4** | Q5 | Q6 | **Q7** | Q8 | Q9 | Q10 | Q11 | Q12 | Q13 | Q14 | **Q15** | Q16 | # of "yes" on critical domains | Quality Category |
| --- | --- | --- | --- | --- | --- | --- | --- | --- | --- | --- | --- | --- | --- | --- | --- | --- | --- | --- |
| Noh, 2023 | Y | **N** | Y | **Y** | Y | Y | **N** | Y | Y | Y | Y | Y | Y | Y | **Y** | Y | 2 | Moderate |
| Howes, 2023 | N | **N** | Y | **Y** | Y | Y | **N** | Y | Y | N | Y | Y | Y | Y | **Y** | Y | 2 | Moderate |
| Piers, 2023 | Y | **N** | Y | **Y** | Y | Y | **N** | Y | Y | N | N | N | N | Y | **N** | Y | 2 | Moderate |
| Wright, 2023 | N | **N** | Y | **Y** | Y | Y | **Y** | Y | Y | N | Y | Y | Y | N | **N** | Y | 3 | Moderate |
| Noh, 2022 | Y | **N** | Y | **Y** | Y | Y | **Y** | Y | Y | N | Y | Y | Y | Y | **Y** | Y | 3 | Moderate |
| Babbage, 2022 | Y | **Y** | Y | **Y** | Y | N | **N** | Y | Y | N | N | N | Y | Y | **N** | Y | 3 | Moderate |
| Cervin, 2022 | Y | **N** | N | **N** | Y | Y | **N** | Y | Y | N | Y | Y | Y | Y | **Y** | Y | 1 | Low |
| Wickersham, 2022 | Y | **Y** | Y | **Y** | Y | Y | **N** | Y | Y | N | Y | Y | Y | Y | **Y** | Y | 3 | Moderate |
| Ivlev, 2022 | Y | **Y** | Y | **Y** | Y | Y | **N** | Y | N | N | N | N | N | Y | **N** | Y | 2 | Moderate |
| Zhou, 2022 | Y | **N** | N | **Y** | Y | Y | **N** | Y | Y | N | Y | Y | Y | Y | **N** | Y | 2 | Moderate |
| Stewart, 2022 | N | **N** | N | **Y** | Y | Y | **N** | Y | Y | Y | N | N | N | N | **N** | Y | 3 | Moderate |
| Reynard, 2022 | Y | **Y** | Y | **Y** | Y | Y | **Y** | Y | Y | N | Y | Y | Y | Y | **Y** | Y | 4 | High |
| Zhou, 2021 | Y | **N** | Y | **Y** | Y | Y | **Y** | Y | Y | N | N | N | Y | Y | **N** | Y | 3 | Moderate |
| Leech, 2021 | Y | **Y** | Y | **Y** | Y | Y | **Y** | Y | Y | N | N | N | Y | Y | **N** | Y | 4 | High |
| Buttazzoni, 2021 | Y | **N** | Y | **Y** | Y | Y | **N** | Y | Y | N | Y | Y | Y | Y | **N** | Y | 2 | Moderate |
| Dewa, 2021 | Y | **Y** | N | **Y** | Y | Y | **N** | Y | Y | N | Y | N | N | Y | **N** | Y | 3 | Moderate |
| Kothgassner, 2021 | Y | **Y** | Y | **Y** | Y | N | **Y** | Y | Y | N | N | N | N | N | **N** | Y | 4 | High |
| Halldorsson, 2021 | Y | **Y** | Y | **Y** | Y | Y | **Y** | Y | N | N | N | N | N | N | **N** | Y | 3 | Moderate |
| Forte, 2021 | Y | **N** | N | **Y** | Y | Y | **N** | Y | Y | N | N | N | Y | N | **N** | Y | 2 | Moderate |
| Christ, 2020 | N | **Y** | Y | **Y** | Y | Y | **N** | Y | Y | N | Y | Y | Y | Y | **Y** | Y | 3 | Moderate |
| Punukollu, 2019 | Y | **N** | Y | **Y** | N | N | **N** | Y | Y | N | N | N | N | N | **N** | Y | 2 | Moderate |
| Rasing, 2019 | N | **N** | Y | **Y** | N | N | **Y** | Y | N | N | N | N | N | N | **N** | Y | 2 | Moderate |
| Grist, 2019 | Y | **N** | Y | **Y** | Y | Y | **N** | Y | Y | N | Y | Y | Y | Y | **Y** | Y | 2 | Moderate |
| Garrido, 2019 | Y | **N** | Y | **N** | Y | Y | **N** | Y | Y | N | Y | Y | Y | Y | **N** | Y | 1 | Low |
| Babiano-Espinosa, 2019 | Y | **Y** | Y | **Y** | Y | Y | **N** | Y | Y | N | N | N | N | N | **N** | Y | 3 | Moderate |
| Barnes, 2018 | Y | **N** | Y | **Y** | N | N | **N** | Y | Y | N | N | N | N | Y | **N** | Y | 1 | Low |
| Dubad, 2018 | N | **N** | Y | **Y** | Y | N | **N** | Y | Y | N | N | N | N | N | **N** | N | 2 | Moderate |
| Hollis, 2017 | N | **Y** | Y | **Y** | Y | Y | **Y** | Y | Y | N | N | N | Y | N | **N** | Y | 4 | High |
| MacDonell, 2017 | N | **N** | Y | **Y** | N | N | **N** | Y | N | N | N | N | N | N | **N** | Y | 1 | Low |
| Grist, 2017 | N | **Y** | N | **Y** | N | N | **Y** | Y | Y | N | N | N | Y | Y | **N** | Y | 4 | High |
| Podina, 2016 | Y | **N** | Y | **Y** | Y | N | **N** | Y | N | N | Y | N | N | Y | **Y** | Y | 1 | Low |
| Stasiak, 2016 | N | **N** | Y | **N** | N | N | **N** | Y | N | N | N | N | N | N | **N** | N | 0 | Low |
| Vigerland, 2016 | N | **N** | N | **Y** | Y | Y | **Y** | Y | N | N | Y | N | N | N | **Y** | Y | 2 | Moderate |
| Struthers, 2015 | Y | **N** | N | **Y** | Y | Y | **N** | Y | Y | N | N | N | Y | N | **N** | Y | 2 | Moderate |
| Pennant, 2015 | Y | **Y** | Y | **Y** | Y | Y | **N** | Y | Y | N | Y | Y | Y | Y | **N** | Y | 3 | Moderate |
| Clarke, 2015 | N | **N** | Y | **Y** | Y | N | **N** | Y | Y | N | Y | N | Y | N | **N** | Y | 2 | Moderate |
| Rooksby, 2015 | Y | **N** | N | **Y** | Y | Y | **Y** | Y | Y | N | Y | Y | Y | Y | **N** | Y | 3 | Moderate |
| Ebert, 2015 | Y | **Y** | Y | **Y** | Y | Y | **N** | Y | Y | N | Y | Y | Y | Y | **Y** | Y | 4 | High |
| Rice, 2014 | Y | **N** | N | **Y** | Y | Y | **N** | Y | N | N | N | N | N | N | **N** | Y | 1 | Low |
| Ye, 2014 | Y | **Y** | Y | **Y** | Y | Y | **N** | Y | Y | N | Y | Y | Y | Y | **N** | Y | 3 | Moderate |
| Boydell, 2014 | N | **N** | N | **Y** | Y | N | **N** | Y | N | N | N | N | N | N | **N** | Y | 1 | Low |
| Seko, 2014 | Y | **Y** | N | **Y** | N | N | **N** | Y | N | N | N | N | N | N | **N** | Y | 2 | Moderate |
| Reyes-Portillo, 2014 | Y | **N** | N | **Y** | Y | Y | **N** | Y | N | N | Y | N | N | Y | **N** | Y | 1 | Low |
| Adelman, 2014 | N | **N** | Y | **N** | N | N | **N** | Y | N | N | Y | N | N | Y | **Y** | Y | 0 | Low |
| Slone, 2012 | N | **N** | Y | **Y** | N | N | **Y** | N | N | N | N | N | N | N | **N** | N | 2 | Moderate |
| Siemer, 2011 | Y | **N** | Y | **Y** | N | N | **N** | Y | N | N | N | N | N | N | **N** | Y | 1 | Low |
| Richardson, 2010 | N | **N** | Y | **Y** | Y | Y | **Y** | Y | N | N | N | N | N | N | **N** | Y | 2 | Moderate |
| PesÃmaa, 2004 | Y | **N** | Y | **Y** | N | N | **N** | Y | Y | N | N | N | N | N | **N** | N | 2 | Moderate |
|  |  |  |  |  |  |  |  |  |  |  |  |  |  |  |  |  |  |  |
| Total # of "Yes" | 32 | **16** | 35 | **44** | 37 | 31 | **14** | 47 | 33 | 2 | 21 | 16 | 24 | 26 | **12** | 44 |  |  |
| Total # of "No" | 16 | **32** | 13 | **4** | 11 | 17 | **34** | 1 | 15 | 46 | 27 | 32 | 24 | 22 | **36** | 4 |  |  |

**Assessing the Methodological Quality of Systematic Reviews (AMSTAR) questions (with bold text indicating critical domains)**

Q1. Did the research questions and inclusion criteria for the review include the components of PICO?

***Q2.*** Did the report of the review contain an explicit statement that the review methods were established prior to the conduct of the review and did the report justify any significant deviations from the protocol?

Q3**.** Did the review authors explain their selection of the study designs for inclusion in the review?

***Q4.*** Did the review authors use a comprehensive literature search strategy?

Q5. Did the review authors perform study selection in duplicate?

Q6. Did the review authors perform data extraction in duplicate?

***Q7.*** Did the review authors provide a list of excluded studies and justify the exclusions?

Q8. Did the review authors describe the included studies in adequate detail?

Q9. Did the review authors use a satisfactory technique for assessing the risk of bias (RoB) in individual studies that were included in the review?

Q10. Did the review authors report on the sources of funding for the studies included in the review?

Q11. If meta-analysis was performed did the review authors use appropriate methods for statistical combination of results?

Q12. If meta-analysis was performed, did the review authors assess the potential impact of RoB in individual studies on the results of the meta-analysis or other evidence synthesis?

Q13. Did the review authors account for RoB in individual studies when interpreting/ discussing the results of the review?

Q14. Did the review authors provide a satisfactory explanation for, and discussion of, any heterogeneity observed in the results of the review?

***Q15.*** If they performed quantitative synthesis did the review authors carry out an adequate investigation of publication bias (small study bias) and discuss its likely impact on the results of the review?

Q16. Did the review authors report any potential sources of conflict of interest, including any funding they received for conducting the review?

**Joanna Briggs Institute Checklist for Systematic Reviews and Research Syntheses**

| First Author (year) | Q1 | Q2 | Q3 | Q4 | Q5 | Q6 | Q7 | Q8 | Q9 | Q10 | Q11 | **# of "yes"** | **Quality Category** |
| --- | --- | --- | --- | --- | --- | --- | --- | --- | --- | --- | --- | --- | --- |
| Ramshaw, 2023 | N | Y | Y | Y | N | N | N | Y | N | Y | Y | 6 | Moderate |
| McCashin, 2019 | N | Y | Y | Y | Y | N | Y | Y | N | Y | Y | 8 | Moderate |
| Ridout, 2018 | N | Y | Y | Y | N | N | N | Y | N | Y | Y | 6 | Moderate |

Q1. Is the review question clearly and explicitly stated?

Q2. Were the inclusion criteria appropriate for the review question?

Q3. Was the search strategy appropriate?

Q4. Were the sources and resources used to search for studies adequate?

Q5. Were the criteria for appraising studies appropriate?

Q6. Was critical appraisal conducted by two or more reviewers independently?

Q7. Were there methods to minimise errors in data extraction?

Q8. Were the methods used to combine studies appropriate?

Q9. Was the likelihood of publication bias assessed?

Q10. Were recommendations for policy and/or practice supported by the reported data?

Q11. Were the specific directives for new research appropriate?
